# Commentary: From the Phenomenology to the Mechanisms of Consciousness: Integrated Information Theory 3.0

**DOI:** 10.3389/fpsyg.2018.00101

**Published:** 2018-02-06

**Authors:** Marek Pokropski

**Affiliations:** Department of Philosophy and Sociology, University of Warsaw, Warsaw, Poland

**Keywords:** consciousness, mechanism, phenomenology, naturalism, phenomenology of consciousness

Mechanistic explanations are applied widely in life sciences (e.g., Craver and Derden, 2013). Interestingly, in recent years there have been attempts at applying the mechanistic approach to cognitive sciences (e.g., Craver, 2007; Bechtel, 2008; Miłkowski, 2016) including an attempt to mechanistically explain consciousness. Masafumi Oizumi, Larissa Albantakis and Giulio Tononi proposed an interesting mechanistic explanation of consciousness in their paper (Oizumi et al., 2014), which is a third version of Integrated Information Theory (IIT) of consciousness, previously formulated by Tononi (2004) and Balduzzi and Tononi (2009). The main hypothesis of IIT is that “consciousness has to do with the capacity to integrate information” (2004, p. 2).

Most of the mechanistic explanations in cognitive sciences begin either from empirical study of existing mechanisms, in this case neural mechanisms (e.g., Crick and Koch, 2003), or from functional analysis of mind/consciousness (e.g., Cummins, 1975) and then move to search for brain mechanisms. What Tononi and colleagues propose is different and novel—their starting point is phenomenology. The methodological idea behind this approach is to identify the fundamental properties of experience and then formulate postulates, which can be considered heuristics that guide the search for physical mechanisms which generate such experience. According to Tononi (2004) the key phenomenological properties of consciousness are differentiation of a large number of experiences and integration of these experiences in a unity. In the later paper by Oizumi et al. (2014), there are more than two fundamental phenomenological properties, represented in a set of phenomenological axioms. These axioms are “self-evident” truths about consciousness, which “cannot be doubted and do not need proof” (p. 2). These axioms are: (i) existence (consciousness exists), (ii) composition (consciousness is structured, i.e., “it consists of multiple aspects”), (iii) information (consciousness is informative, i.e., “each experience differs in its particular way from other possible experiences”), (iv) integration (consciousness is integrated in non-reducible way to its components), and (v) exclusion (“each experience excludes all others—at any given time there is only one experience having its full content”) (2014, p. 2–3).

It is important to emphasize that the paper by Oizumi et al. is very rich and it is impossible to comment here the whole theory. I also agree with their approach to begin with phenomenology rather than neural mechanisms. That is why I discuss here the first part of the paper, namely the axioms and postulates concerning physical realization, grounded in these axioms.

Firstly, although some axioms are not controversial, e.g., the first axiom which states that consciousness exists seems obvious, because we have to acknowledge the existence of the phenomenon we want to explain, others are. The fifth axiom of exclusion, according to which we cannot have multiple partial experiences and “each experience has definite borders” (2014, p. 3) seems particularly disputable. It seems we can adduce many counterexamples of experiences with blurred or overlapping borders such as peripheral vision, perception of reversible figures or multitasking. Conscious experiences seem not to be clear-cut entities, but rather dynamic and interrelated processes. More importantly, however, this axiom seems to be inconsistent with the second axiom of composition, which states that in one experience, we can have varied conceptual content, e.g., a half-red half-green circle is moving from left to right. Axiomatic systems cannot be inconsistent, thus either one of these axioms should be rejected or reformulated and inconsistency overcame. Finally, authors derive from the fifth axiom a postulate that “a mechanism can contribute to consciousness at most one cause-effect repertoire, the one having the maximum value of *integration/irreducibility*” (p. 3), which is crucial for the IIT 3.0. However, it might be the case that fifth axiom is incorrect or, even more radically, that postulates about physical mechanisms do not have to follow from phenomenology. Although the claim about structural correspondence between phenomenology and physical realization is necessary for the whole explanatory enterprise, it is definitely not obvious and requires further argumentation.

One can formulate doubts about other axioms as well or propose another such as: subjectivity (every conscious experience is subjective, i.e., it is an experience of a subject), intentionality (consciousness is about something), temporality (consciousness is a temporal process), or qualia (there is a specific qualitative state in experiencing X, i.e., there is “something what it is like” to experience X). However, the question is: are axioms of consciousness really immediately evident as their proponents claim? In the first formulation of IIT from 2004, Tononi himself admits that the information integration property of consciousness “may not seem self-evident” because we take it for granted and it is necessary to employ some thought experiments to apprehend this and other key aspects of subjective experience as self-evident truth (2004, p. 2). However, if that is the case, then discussion of methodological fundaments of such phenomenology and its methods is necessary. It is true that axioms do not need proof in a formal sense, but they may require some justification or illustration.

In grounding phenomenological axioms we may refer to the huge tradition of phenomenological philosophy started by Husserl (e.g., 1913/1983,^1991^) and still being cultivated, also in cognitive sciences (e.g., Varela, 1999; Zahavi, 2005; Thompson, 2007). The objective of Husserlian phenomenology was precisely to elaborate a methodological analysis of consciousness, which was neither introspective (based on subjective inner experience) nor folk (derived from our commonsensical or linguistic intuitions). For example, an interesting insight from Husserlian phenomenology, which may be considered as an axiom of consciousness, especially when we think about consciousness as a process of integration, is temporality. According to Husserl (1991) consciousness in essentially a temporal phenomenon. Furthermore, he describes a specific temporal functions of consciousness, such as retention and protention, which generate a conscious experience. This is obviously just a suggestion and would need a separate essay to show how phenomenological analyses of temporal functions can improve searching for mechanisms of consciousness.

## Author contributions

The author confirms being the sole contributor of this work and approved it for publication.

### Conflict of interest statement

The author declares that the research was conducted in the absence of any commercial or financial relationships that could be construed as a potential conflict of interest.
